# Size Effect of Nanoceria Blended with CIME Biodiesel on Engine Characteristics

**DOI:** 10.3390/nano13010006

**Published:** 2022-12-20

**Authors:** Vivek Pandey, Irfan Anjum Badruddin, Sarfaraz Kamangar, Addisu Bekele Alemayehu

**Affiliations:** 1 Mechanical Engineering Department, School of Mechanical, Chemical and Materials Engineering, Adama Science and Technology University, Adama 1888, Ethiopia; 2 Mechanical Engineering Department, College of Engineering, King Khalid University, Abha 61413, Saudi Arabia

**Keywords:** cerium oxide, nanoparticles, biodiesel, *Calophyllum Inophyllum* methyl ester, performance, emissions

## Abstract

Diesel fuel blends with biodiesels are expected to mitigate the rising price and demand of conventional fuels. Biodiesel fuel blends are also known to reduce engine emissions. Biodiesel is produced from various sources, one of which is *Calophyllum Inophyllum* methyl ester biodiesel (CIMEBD). Even though it serves to mitigate the energy crisis and has a low overall carbon footprint, CIMEBD has certain negative issues relating to engine performance and emission characteristics. Nanoparticle (NP) addition is known to enhance the engine performance characteristics of next generation biofuels. CeO_2_ (cerium oxide or ceria) NPs of varying size are used in this study along with 25:75 biodiesel–diesel (BD) blend and a fixed NP concentration of 90 ppm. Ceria NP-doped fuel is shown to have better engine performance compared to diesel and BD blend for all load conditions. Improvements in brake thermal efficiency (BTE) and brake-specific fuel consumption (BSFC) values equal to +30% and −46%, respectively, are observed from experiments for ceria NP-doped biodiesel, compared to diesel–biodiesel (BD) blend. Ceria NPs in the 20 to 40 nm range have optimum engine performance characteristics. Compared to BD blends, NP-doped biodiesel shows improvements in NOx, CO, CO_2_, UHC, and soot parameters up to −35%, −60%, −35%, −38%, and −40%, respectively. Likewise, the optimum size of ceria NPs is in the range 20–40 nm for better emission characteristics.

## 1. Introduction

Internal combustion (IC) engines are predominantly used energy conversion devices, and they will be in use for a long time to come [1,2]. Among IC engines, diesel engines are mainly used for transport due to low throttling losses, high fuel efficiency, turbocharging capacity, high compression ratio, and the absence of knocking [3,4].

A predominant fraction of the world’s energy needs is obtained from fossil fuels [5]. For example, the transport sector obtains 95% of its conventional energy needs from non-renewable, liquid fossil fuels [6,7]. However, fossil fuels are not expected to last for more than a few decades [8]. Moreover, fossil fuels account for more than 95% of carbon emissions worldwide [9]. It is further expected that there will be a steady increasing demand for liquid fuels in the near future [10]. There is an initiative by the International Energy Agency (IEA) that is aimed at reducing net engine emissions to zero by 2050. In this respect, the reduction of fossil fuel dependence is considered a major challenge. Due to these reasons, various forms of alternative fuels are being investigated, such as syngas [11], biodiesel [12,13], natural gas [14,15], hydrous ethanol [16], and bio-butanol [17].

Biodiesel research occupies a substantial proportion of the literature, and various aspects of biodiesels have been investigated [18,19,20,21,22,23]. A source of biodiesel production is the non-edible oil seed of *Calophyllum Inophyllum*, from the species Clusiaceae [24]. *Calophyllum Inophyllum* trees are available locally in South and South East Asia. They can grow in harsh climates and provide substantially higher oil yield compared to various other sources of biodiesel [25]. Properties of *Calophyllum Inophyllum* methyl ester biodiesel (CIMEBD) account for better engine performance characteristics. Some of the properties of CIMEBD are listed in Table 1. Compared to other biodiesels, CIMEBD exhibits better oxidation stability resulting in a better quality fuel with stable combustion characteristics [26]. It has a low viscosity index and, therefore, better lubrication and cooling characteristics.

The higher cetane number of CIMEBD results in reduced ignition delay and better power and torque characteristics [29]. However, CIMEBD fueled engines have slightly reduced brake thermal efficiency (BTE) and fuel economy [30], which, however, can be reversed by using high injection pressures [31]. The higher oxygen content of CIMEBD is known to enhance combustion characteristics [32].

Nanoparticles (NPs) are particles of metals, metal oxides, or inorganic compounds such as carbon nanotubes (CNT) or carbon nano sheets (CNS), and they usually range from 10–100 nm. They form nano-emulsions with diesel/biodiesel fuel and enhance the fuel properties as well as engine performance and emission characteristics [33]. Improvements in injection timing, enhancement in calorific value, flash point, and fire point occur when NPs are blended with fuel [34,35].

NPs have high surface area, which provides large numbers of reactivity sites resulting in enhanced catalytic activity [36]. They cause a reduction in ignition delay and assist in fast energy release [37]. Therefore, they provide better combustion characteristics [38] and are beneficial for thermal efficiency. They also assist in reduction of harmful engine exhaust and emissions and BSFC in diesel engines [39].

Cerium oxide (ceria) NPs are emerging as a novel NP resource with a wide range of applications. Various methods for the preparation of cerium oxide (ceria) are known to exist. The precipitation method was applied by Fifere et al. [40] using Ce(IV) sulphate as a precursor dispersed in glycerol with varying synthesis parameters such as temperature or precipitating agent. Ranasinghe et al. [41] used a soluble borate glass to produce nanoceria with specific ratios of Ce^3+^/Ce^4+^, via controlled glass-melting parameters. Cerium oxide (CeO_2_) nanoparticles were synthesized with a chemical precipitation method with different experimental conditions using cerium nitrate hexahydrate (Ce(NO_3_)_3_·6H_2_O) as a precursor. Pop et al. [42] synthesized CeO_2_ nanoparticles by a wet chemical synthesis route, using the precipitation method and the simultaneous addition of reactants (WCS–SimAdd). The method of synthesis is known to affect the properties of the nanoceria particles. Ceria or NPs are known to have a variety of uses. They have been demonstrated to act against pathogenic bacteria [42]. Nanostructured ceria has multiple, emerging bio-related applications (Rozhin) [43]. Liu et al. [44] demonstrated that nanostructured ceria-based electrolytes have potential application in low temperature, solid oxide fuel cells. Cerium oxide (ceria) nanoparticles (NPs) are known to be an efficient optical fluorescent material under violet excitation. Shehata et al. [45] used this characteristic of *Ceria NP*s for application as an optical sensor via the fluorescence quenching technique. Various reviews and studies on cerium oxide NPs have indicated that cerium oxide additives enhance the fuel combustion and emission properties. Cerium oxide nanoparticles are known to effectively reduce all emissions of concern, including NOx, unlike many other NPs. Since ceria NPs have a wide and emerging range of applications, they need to be investigated more thoroughly as fuel additives.

The effect of cerium oxide (CeO_2_) NPs along with CIMEBD is, therefore, investigated in this work, keeping in view the enhanced results of NP additives to biodiesels. Review on the literature relating to the use of CIMEBD and cerium oxide NPs is presented in the following section, which will highlight the fact that there are relatively few works that have dealt with cerium oxide NPs in combination with CIMEBD, which is a proven and prominent source of biodiesel in South Asia. In addition, there have been hardly any investigations that present smoke emission results with the above-mentioned combination, namely CIMEBD and cerium oxide NPs.

The structure of the article is divided as follows: Section 1.1 deals with the literature review relating to cerium oxide NPs and CIME biodiesel. Section 2 provides materials and methods used in the study. Section 2.1 deals with cerium oxide NP synthesis and characterization. Section 2.2 details CIME biodiesel synthesis, whereas Section 2.3 has the experimental details and procedure followed in the investigation. Section 3 is for results and discussion, followed by the conclusions.

### 1.1. Literature Review

This section reviews some of the relevant and recent literature relating to CIMEBD biodiesel, and the engine characteristics with use of CeO_2_ NPs are also reported. It is found from the literature presented below that only one relevant work exists for CIMEBD with ceria NP investigation [46] (Table 2). Moreover, none of the mentioned literature has presented smoke emissions data.

Some of the recent and relevant review papers for biodiesel-based blends with NP additives are by Lv et al. [47], Haq et al. [48], and Ampah et al. [49]. Lv et al. [47] reviewed the effects of nano-additives added to diesel–biodiesel fuel blends on combustion and emission characteristics of diesel engines.

Various studies in their review mention that adding cerium oxide or ceria nanoparticles to diesel–biodiesel fuel blends can improve combustion and reduce emissions to varying degrees. Haq et al. [48] reviewed fuel additives, including nano-additives and their spray characteristics, for diesel-based fuel blends. They confirmed that cerium oxide nanoparticles have high surface/volume ratio and cause improvement in chain reactions during combustion. Ampah et al. [49] reviewed the progress and recent trends in the application of nanoparticles as low carbon fuel additives. However, their review does not have results for the effect of cerium oxide NP additives on NOx emissions.

There are relatively few investigations relating to CIMEBD blended with NPs. NPs can improve the relatively low brake thermal efficiency of CIMEBD due to better combustion characteristics arising from the enhanced oxidation reaction and heat release rate [50]. Air–fuel mixture formation is assisted by the ‘micro-explosion’ of NPs [51]. This aids in reducing the BSFC for engines fueled with NP-blended CIMEBD compared to the diesel–biodiesel blend (BD) [46]. The excess oxygen supplied by metal oxide NPs is an aid to combustion due to a higher concentration of locally available oxygen [51]. Even though the lower calorific value of CIMEBD results in a lower overall energy release, its higher cetane number results in reduced ignition delay, increase in peak cylinder pressures, and heat release rate (HRR). These results are further improved with NP-blended CIMEBD [51]. The activation energy of NPs suppresses the deposition of non-polar particles on the combustion chamber wall, thereby reducing hydrocarbon formation [46]. Metal oxide NPs aid in the decrease in carbon monoxide (CO) emissions since they are oxygen carriers, and this fact explains the efficient conversion of carbon monoxide (CO) into carbon dioxide (CO_2_). Very few research works dealing with 100% biodiesel are found in the literature. Out of these, the one by Ashok et al. [52] is prominent. However, the authors have not studied the effect of NPs on smoke emissions. Smoke investigations with ceria NP combined with CIMEBD are also lacking in the literature. Kumar et al. [38] tested cooking oil biodiesels blended with diesel along with 0.008 wt.% ceria (CeO_2_) NPs. A 15.9%, 11.8%, and 5.9% reduction in UHC, nitrogen oxides, and smoke were observed. The reductions were more prominent for higher concentrations of biodiesel and NPs. Kukucosman et al. [53] demonstrated that 0.5 wt.% of NPs provides the best spray characteristic enhancement, while 2–2.5 wt.% of NPs was good for enhanced combustion characteristics. Similarly, 0.01 wt.% TiO_2_ NPs added to diesel-biodiesel-n-butanol blends had the best combustion and emission characteristics in a direct injection CI engine [54].

NOx emissions generally increase with NP concentration, with the exception of ceria NPs, in which case the NP surface acts as a catalytic convertor and breaks down NOx into oxygen and nitrogen. Ooi et al. [55] assessed diesel engine emissions with graphite oxide (GO), single walled carbon nanotubes (SWCNT), and ceria NP additives to diesel. Higher NOx emissions were observed with 9% GO and 15% SWCNT due to higher cylinder temperatures. The opposite effect was observed with ceria NPs. Khan et al. [34] demonstrated that ceria NPs provide effective NOx reduction capability due to their unique catalytic property.

Similarly, Hawi et al. [56] experimentally investigated the performance of a compression ignition engine fueled with waste cooking oil biodiesel–diesel blend enhanced with iron-doped cerium oxide NPs. Hossain and Hussain [57] studied the impact of CeO_2_ and Al_2_O_3_ nano-additives on the performance and combustion characteristics of neat jatropha biodiesel. Hussain et al. [58] investigated the enhancement in combustion, performance, and emission characteristics of a diesel engine fueled with 3% Ce-coated ZnO NP additives added to soybean biodiesel blends.

There are various papers on nanoparticle additives to biodiesel that mention the use of CeO_2_ NPs. However, none of these provide results for engine and emission characteristics with a combination of CIMEBD with CeO_2_ NP, and this may be confirmed from the recent and prominent papers provided in Table 2. In this study, a diesel engine fueled with CIMEBD with ceria NP additives is investigated for engine performance and emission characteristics.

**Table 2:** Summary of investigations using pure (unmixed) CeO_2_ NPs with relevant details; —indicates that the values are not provided.

S.No.	Ceria NP Size (nm)	NP Concentration	Biodiesel–Diesel Blend *	Reference	Year
1	-	50, 100 ppm	Jatropha biodiesel	[57]	2019
2	25	20,40,60 ppm	CIMEBD	[46]	2016
3		90 ppm	WDE-RMEBD	[59]	2018
4		90 ppm	Water + D + Bd	[60]	2018
5	10–16	25 ppm	GMGBD	[61]	2019
6	50	80 ppm	WCOBD + D-20:80	[62]	2019
7	32, 36	100 ppm	GSOBD	[63]	2019
8	50		LGO + DEE + D	[64]	2016
9	10–20	30 ppm mass	LGO + Water + D	[65]	2019
10	5–10; 10–20	50 ppm	Karanja MEBD (Pongamia)	[66]	2019
11	25	50,100,150 ppm	Mahua MEBD	[67]	2019
12	16		LGOBD	[68]	2016
13	10,30,80	80 ppm	WCOBD + D-20:80	[69]	2021

* WCO-Waste cooking oil, BD-Biodiesel, D-Diesel, RMEBD-Rapeseed methyl ester biodiesel, GSOBD-Grapeseed oil biodiesel, LGO-Lemongrass oil, MEBD-Methyl ester biodiesel.

Table 2 provides a summary of the various recent, relevant, and prominent (with high journal impact factor) works using pure (unadulterated) CeO_2_ NPs alone. It can be seen that none of the works except the one by Vairamuthu et al. [46] contain the investigation of ceria NP added to CIMEBD. Moreover, none of the works mentioned in the literature mention smoke emission characteristics, which are an important emission parameter. Therefore, this gap in the literature, namely the study on ceria NP additives to CIME biodiesel and related engine performance and emissions, is reported in this paper.

## 2. Materials and Methods

CeO_2_ nanoparticle and CIMEBD synthesis and characterization are detailed in this section. The experimental setup, instrumentation, and operating conditions are also elaborated.

### 2.1. Cerium oxide NP Synthesis and Characterization

**Figure 1:**
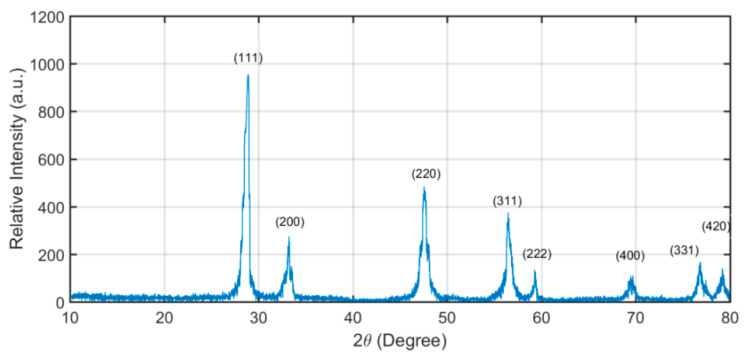
XRD intensity versus 2θ indicating ceria NPs.

Cerium oxide NPs were manufactured and characterized by Sigma Aldrich^®^. Mechanical vibration was applied at 46 kHz, and also the surfactant cetrimonium bromide ([(C_16_H_33_)N(CH_3_)_3_]Br), or CTAB, was used for inducing a stable suspension of NPs in the fuel. Figure 1 displays the X-ray diffraction spectra for ceria NPs. CuKα radiation of 0.15 nm wavelength was used for the characterization. Diffraction peaks for the NPs indicate the presence of ceria as per Standards of the Joint Committee for Powder Diffraction Studies (JCPDS) File No. 34-0394. The average NP size was found from Scherrer’s [70] equation.

### 2.2. CIME Biodiesel Synthesis

CIMEBD was acquired from a local vendor, Sigma Aldrich Pvt. Ltd., Mumbai, India. The process for CIMEBD manufacture is described. Oil seeds of *Calophyllum Inophyllum* are dried and then screw pressed for oil extraction; the resulting raw oil is dark green in color. Among the various prevalent methods, trans-esterification is used for oil extraction as it offers high quality raw oil yield in a relatively short time, with the resulting biodiesel having properties as per ASTM D 6751 standards [71]. During trans-esterification, raw seed oil is broken down into esters and glycerol with the help of an alcohol and a catalyst. Since the free fatty acid (FFA) content of the raw oil is more than 4%, a two-stage esterification process is applied [72]. Triglycerides are converted to diglycerides by acid-esterification. Thereafter, alkali-based esterification results in low-density ester and high-density glycerol. The two compounds are immiscible and form separate layers. The esters are the primary constituents of the biodiesel.

### 2.3. Experimental Details and Procedure

**Figure 2:**
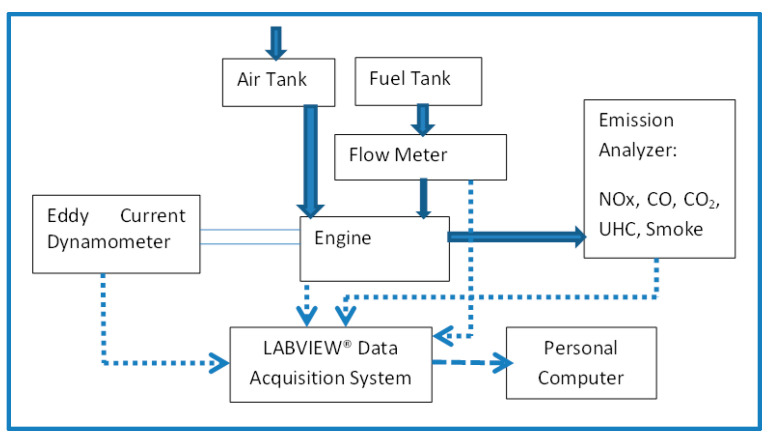
Schematic of experimental rig.

A naturally aspirated, 4-stroke, single cylinder, diesel research engine equipped with an eddy-current dynamometer is used. Figure 2 shows schematic of the experimental setup. Table 3 displays the relevant engine specifications. Horiba^®^ emission analyzer modules were employed to measure the tailpipe emissions (HC, CO, CO_2_, and NOx). The test rig is integrated with the instrumentation for acquisition of load, temperatures, airflow, crank angle (CA), and combustion pressure. Data is acquired by the LabView^®^ data acquisition (DAQ) system. A fixed ratio of diesel–biodiesel blend in the ratio 25:75 is chosen while varying the size of ceria NPs. The ceria NP concentration in our study is fixed at 90 ppm, which is the same as the optimized NP size from various earlier works, as reported by Hawi et al. [56]. The fuel blend details are shown in Table 4.

Root mean square (RMS) error of measurements is used for estimating the measurement uncertainty from the following equation:

$$e_{R}={\left[{\left(\frac{\partial f}{\partial x_{1}}e_{1}\right)}^{2}+{\left(\frac{\partial f}{\partial x_{2}}e_{2}\right)}^{2}+\cdots +{\left(\frac{\partial f}{\partial x_{n}}e_{n}\right)}^{2}\right]}^{\frac{1}{2}}$$

where e_R_ is the uncertainty in result R, and x_i_ are measured variables. The emission measurement uncertainties (as per manufacturer specifications) are shown in Table 5.

## 3. Results and Discussion

**Figure 3:**
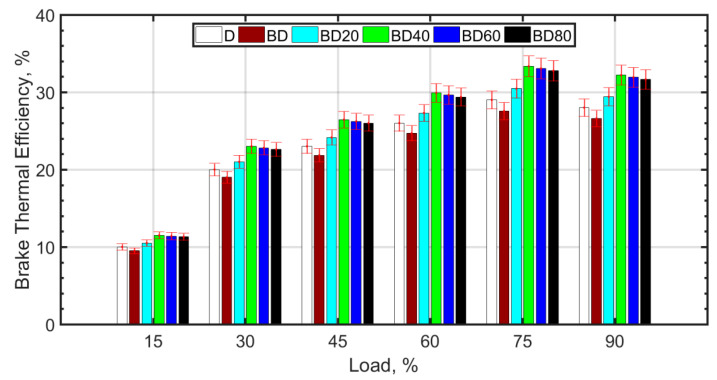
Brake thermal efficiency variation with load.

Figure 3 shows the brake thermal efficiency (BTE) with load for the fuel blends.

**Figure 4:**
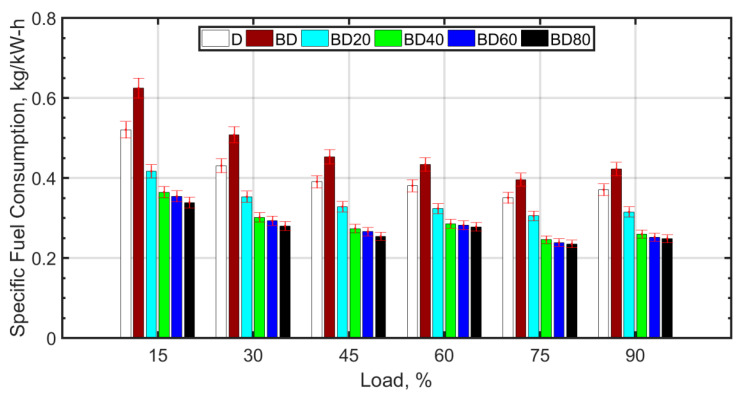
Specific fuel consumption variation with load.

The BTE increases with load for all the fuel blends. This is expected since the cylinder temperature increases with load due to the higher mass of fuel injected, thereby causing higher cylinder temperatures. For loads greater than 75%, there is no significant change in BTE, because the fuel becomes richer, and there is a tradeoff between higher combustion temperature and combustion inefficiency. The BTE trend across the fuel blends is similar for all loads. The BTE is lower for BD compared to diesel (D). Some authors, such as Vairamuthu et al. [46], have attributed this to the lower calorific value [73], or density [74,75] of CIMEBD, whereas others speculate that the higher molecular oxygen content of the biodiesel may be the reason for the lower BTE [32]. However, for the same load, a higher mass of biodiesel injection is required as compared to diesel. The reason for the slightly reduced BTE in the case of BD is more likely due to the higher biodiesel viscosity [76,77] and surface tension, which cause relatively inferior atomization, delayed ignition, and relatively inefficient combustion [38]. Ceria NPs assist in better atomization due to lower ignition delay caused by micro-explosion, therefore, more efficient combustion and better BTE [51]. Biodiesel blends with ceria NPs perform better in terms of BTE compared to diesel. The optimum BTE values are obtained for BD40, that is, for biodiesel blended with 40 nm ceria NPs. It is observed from other studies that ceria NPs between 30 nm and 40 nm size are most desirable for the purpose of improving the BTE [38]. The NPs have high surface-to-volume ratio and assist in better atomization due to micro-explosion and micromixing [78]. However, smaller NPs tend to agglomerate, and this leads to higher fuel viscosity [79] and poor atomization. It is also known that agglomeration of NPs results in a wider NP size distribution and deterioration of the desirable NP properties. On the other hand, larger NPs are known to decrease the thermal conductivity of the nanofuel. It is also known that it is difficult to maintain the stability of large sized NPs in the base fuel. Therefore, the BTE increase for BD60 and BD80 is comparatively low. Figure 4 shows the brake specific fuel consumption (SFC) variation with load for the fuel blends.

SFC decreases with an increase in load for all fuel blends. It attains a minimum for 80% load and thereafter increases slightly. With an increase in load, a reduction in SFC is observed due to more efficient combustion as a result of higher temperatures. SFC for BD is 10–20% more when compared to diesel. The biodiesel blend (BD) has a 15–20% lower calorific value compared to diesel; this requires that more fuel on mass basis should be injected into the cylinder for a given load. In addition, the kinematic viscosity of BD at 100 °C is almost twice that of diesel; this deteriorates the atomization of the fuel. This leads to larger ignition delay and poorer combustion efficiency. With the addition of ceria NPs, the combustion characteristics are observed to improve. This can be explained based on the ‘microexplosion’ phenomenon [51,79] of the NPs, which aids in superior atomization of fuel droplets and better localized mixing of the fuel and air. The molecular oxygen that is contained in the NPs also assists in increasing the combustion efficiency. The oxygen contained in BD aids in enhancing combustion but only after effective fuel atomization and air–fuel mixing occur.

**Figure 5:**
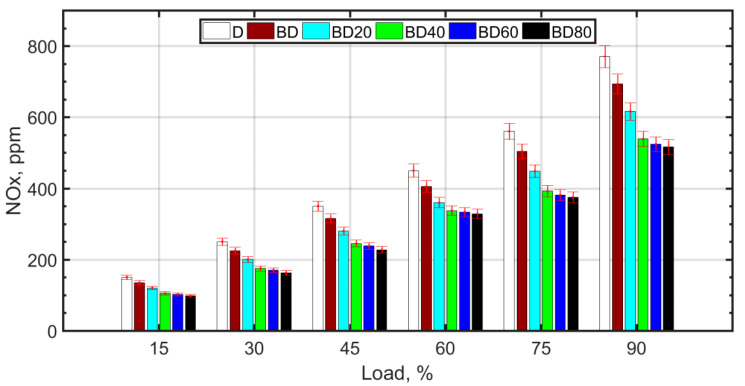
NOx variation with load.

With the increase in NP size, it is observed that the SFC is slightly higher. It has been shown that larger sized NPs have lower thermal conductivity [69] and higher fuel viscosity. The lower thermal conductivity and higher viscosity of larger NPs cause poor atomization, mixing, and combustion characteristics, which increases the SFC. Lower sized particles are known for higher agglomeration and, therefore, higher viscosity of the nanofuel. However, this can be reduced by means of surfactants [80], which can be a future avenue of research. Figure 5 shows the NOx variation with load for the fuel blends.

The Zeldovich mechanism is a well-known route for NOx formation, and the primary constituent of NOx is ‘thermal NOx’, which has a strong dependance on the combustion temperature [81]. Pure biodiesel blend (BD) has inferior atomization due to higher viscosity and lower calorific value, resulting in lower adiabatic flame temperature with biodiesel; both these factors result in a lower in-cylinder peak temperature, resulting in lower NOx formation. With the addition of ceria NPs, the combustion efficiency is enhanced due to the ‘microexplosion’ of the NPs, resulting in superior atomization, air–fuel mixing, and reduced ignition delay. This increases the peak cylinder temperatures, which should result in higher NOx formation. However, comparatively high NOx values are reported with NPs other than cerium oxide in the literature. The explanation for this is attributed to the NOx reducing property of cerium oxide NPs [78]. The lowest NOx values are observed for BD40. For larger sized NPs, the higher fuel viscosity due to NP agglomeration causes poor atomization, and the lower surface-to-volume ratio results in lower thermal conductivity and relatively inferior combustion compared to BD20 and BD40. Therefore, the NOx comparative reduction is not significant for larger sized NPs. Furthermore, for BD60 and BD80, the slight advantage of lower NOx is lost because of the lower BTE and higher SFC with larger sized NPs in the fuel.

**Figure 6:**
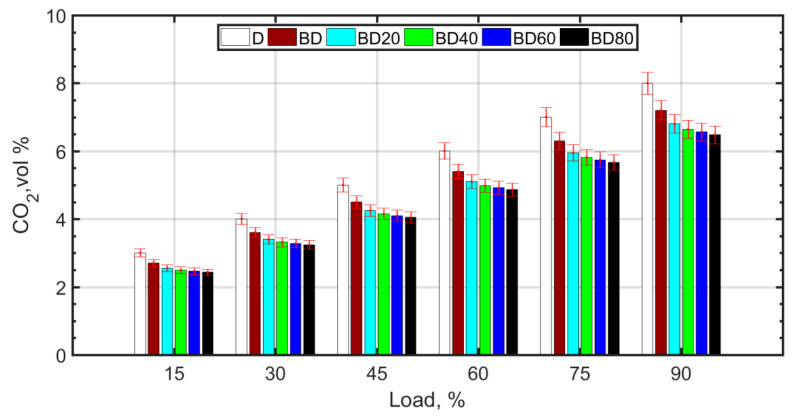
CO_2_ variation with load.

**Figure 7:**
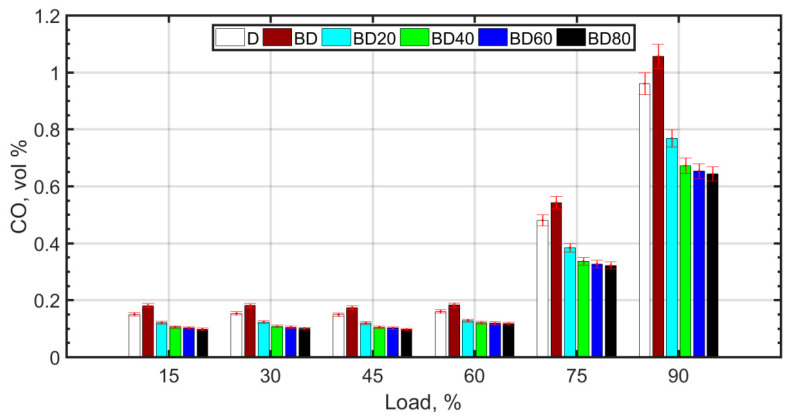
CO variation with load.

The reversible reaction involving combination of carbon monoxide and atomic oxygen is primarily responsible for CO_2_ formation (Figure 6). CO_2_ and CO are negatively correlated, that is, the greater the amount of CO_2_, the less CO formation occurs. Usually, for higher temperatures and pressures, the forward reaction, as shown below, is favored:

CO + O → CO_2_,

whereas lower temperatures and pressures favor the formation of CO as a result of poor combustion efficiency. The higher viscosity of BD and the resulting poor atomization causes retarded droplet evaporation. It is known that droplet evaporation time is directly proportional to the droplet diameter. This causes retarded ignition and inefficient combustion for BD. With the addition of NPs, combustion efficiency improves due to micro-explosion and the oxygen carried by the NP molecules. There is a slight decrease in CO_2_ and a corresponding increase in CO. For the fuel blends with NPs, there is complete combustion due to micro-explosion, and reduced ignition delay. However, the molecular oxygen donated by ceria NPs and also the molecular oxygen of the biodiesel results in an overall reduction in CO_2_ emissions. However, CO formation is slightly enhanced for the diesel–biodiesel blend (BD), as shown in Figure 7. CO formation is heightened at higher loads due to rich mixtures at loads greater than 75%.

**Figure 8:**
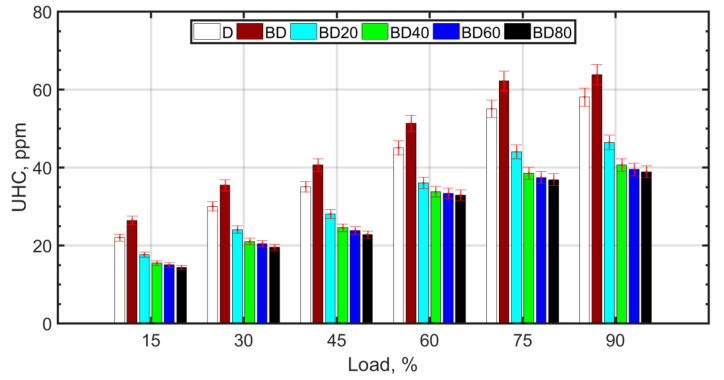
UHC variation with load.

The reasons for the formation of UHCs are similar to those of CO, as shown in Figure 8. High temperature, pressure, and sufficient oxygen cause a reduction in UHCs and an increase in CO_2_. For larger sized NPs, it is known that the nanofluid has lower thermal conductivity. In addition, larger NPs result in issues such as unstable fuel suspension and fuel injector clogging.

**Figure 9:**
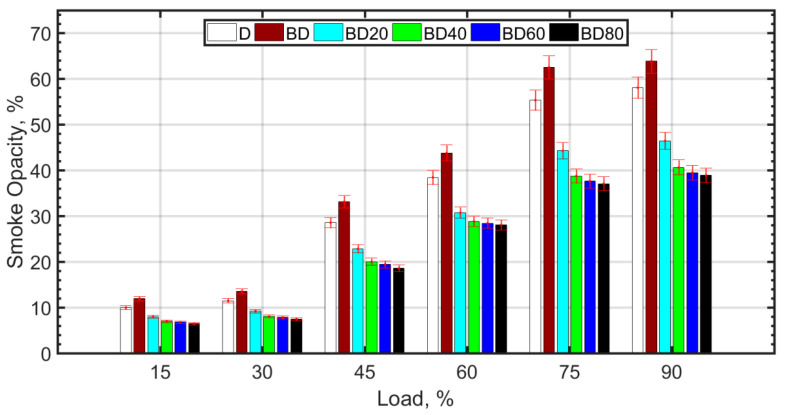
Smoke opacity variation with load.

Smoke/soot trends are shown in Figure 9. Soot formation mechanism is different and more complex from the oxidation of carbon species such as CO and UHC. The reversal reaction as in the case of CO and CO_2_ is not applicable for soot. Soot formation requires the presence of soot precursors such as naphthalenes [82] and aromatics in the fuel. Since BD has higher aromatic content [26] compared to diesel, there is an increase in smoke opacity for BD, which, however, decreases with NP addition. Large NP size does not seem to have much effect on smoke opacity reduction compared to 40 nm NP size. The reasons may be due to reduced effectiveness of larger sized NP due to agglomeration, higher fuel viscosity, poor atomization, and lower temperatures that can inhibit the oxidation of large chain soot particles. In general, better combustion characteristics of fuels due to NPs are caused by good atomization, molecular oxygen, lower ignition delay, and related phenomenon [53].

## 4. Conclusions

It is known that NP additives can enhance engine performance and reduce emissions. A wide variety of nanoparticles have been used for this purpose. The use of cerium oxide NP is of interest, because, unlike other NP additives to fuel, they can reduce NOx emissions in combination with providing enhanced BTE. Investigations with pure cerium oxide nanoparticles are few in number. Another motive for the research is the application of CIME biodiesel, which is sourced from the non-edible seeds of *Calophyllum Inophyllum*, and which are found abundantly in South Asia. However, the combined effect of CIMEBD with ceria NP is scarcely available in the literature. Furthermore, soot is an important emission of concern, and the soot emission results are also not available for NP additives to diesel or biodiesel blends. We investigate the effects of different sized ceria NPs as additives to CIMEBD blended with diesel. Experiments were conducted with *Calophyllum Inophyllum* methyl ester biodiesel (CIMEBD) + diesel in the ratio 25:75 blended and cerium oxide (ceria) NPs of sizes, 20, 40, 60, and 80 nm. Ceria concentration of 90 ppm by mass is employed in the investigation. The general trend of variation for BTE, BSFC, and regulated emissions such as NOx, CO, CO_2_, UHC, and soot for differing engine load conditions reveal similarities with previous literature. Ceria NP-doped fuel demonstrates better engine performance and emission characteristics, including NOx emission reduction, which is the major point of difference while using ceria NPs. More specific conclusions are mentioned in the succeeding paragraph.

Ceria NP-doped fuel performs better over diesel and pure biodiesel for all load conditions. Improvement in BTE and BSFC values equal to +30% and −46%, respectively, are observed for ceria NP-doped biodiesel, compared to pure biodiesel. Ceria NPs sizes ranging from 20 nm to 40 nm offer optimum performance characteristics.

Similar to the performance parameters, improvement in NOx, CO, CO_2_, UHC, and soot parameters up to −35%, −60%, −35%, −38%, and −40%, respectively, are observed while comparing NP-doped biodiesel and pure biodiesel. Similar to the performance parameters, the optimum size of ceria NPs is in the range 20–40 nm for desirable emission characteristics.

Limitations of this work include insufficient evaluation of the toxicity of the NP to humans from their inevitable emissions as engine exhaust. Additionally, different morphologies or texture of the NP has not been studied in this respect. This may be achieved by using nanostructured ceria-based catalysts for combustion application to diesel engines, which will be a future avenue of research. In general, for biodiesel research with NP additives, the morphology or structure effect of the NP has not been investigated. It is expected that differently textured NPs or nano structured ceria would have better properties compared to simply prepared NPs, whose texture or morphology has not been controlled at the nano scale. This avenue of research holds immense promise for the case of diesel–biodiesel blends with ceria NP or other NP additives, in the context of engine performance enhancement and emission reduction

## Figures and Tables

**Table 1:** Properties of CIME [26,27,28].

Property	Diesel	CIMEBD
Kinematic Visosity@100 °C	2.69	4.72–5.68
Flash Point	68.5	140–165.5
Density@40 °C	839.6	868–877
Lower Calorific Value (kJ/kg)	45,304	38,330–39,513
Oxidation Stability (hrs. at 100 °C)	-	3.58–14.27
Cetane Number	51.7	55–63

**Table 3:** Engine Specifications.

Engine Specifications	
Number of Cylinders	1
Stroke	114.3 mm
Bore	82.5 mm
Swept Volume	612 cc
Fuel System	Direct Injection
Compression Ratio	(16:1)
Cooling System	Water cooled

**Table 4:** Fuel Blends and Nomenclature.

Fuel Blend Nomenclature	D	BD	BD20	BD40	BD60	BD80
Fuel Blend Specification	Diesel	25% CIMEBD, 75% Diesel	BD + NP (20 nm)	BD + NP (40 nm)	BD + NP (60 nm)	BD + NP (80 nm)

**Table 5:** Instrument Range/Accuracy.

Instrument Range/Accuracy/Uncertainty	
Load cell dynamometer	0–1200 Nm; ±0.25% of full scale
Coriolis flow meter	0–240 kg/h; ±0.1% of measured value
K-type thermocouple	±0.75%; −200–1250 deg. C
Horiba exhaust analyzer	CO: 0.02% (*v*/*v*); −0.2% to +0.2% uncertainty
	HC: 1 ppm vol. (0−2000 ppm vol.); −1.3% to +1.3% uncertainty
	O_2_:10 ppm vol. (2000–10,000 ppm vol.)
	CO_2_: 0.02% (*v*/*v*); −0.2% to +0.2% uncertainty
	NO: 1 ppm vol.; −1.3% to +1.3% uncertainty

## Data Availability

All the data is provided within the manuscript.
